# Purple sweet potato color protects against hepatocyte apoptosis through Sirt1 activation in high-fat-diet-treated mice

**DOI:** 10.29219/fnr.v64.1509

**Published:** 2020-02-04

**Authors:** Weitong Su, Cheng Zhang, Feng Chen, Junwen Sui, Jiaqi Lu, Qingqing Wang, Qun Shan, Guihong Zheng, Jun Lu, Chunhui Sun, Shaohua Fan, Dongmei Wu, Zifeng Zhang, Yuanlin Zheng

**Affiliations:** Key Laboratory for Biotechnology on Medicinal Plants of Jiangsu Province, School of Life Science, College of Health Science, Jiangsu Normal University, Xuzhou, Jiangsu Province, P. R. China

**Keywords:** purple sweet potato color, hepatic apoptosis, Sirt1, P53, Akt, high-fat diet

## Abstract

**Background:**

Recent evidence indicates that the inhibition of hepatocyte apoptosis is possible to develop a potential therapeutic strategy for nonalcoholic fatty liver disease (NAFLD). Our previous work suggested that purple sweet potato color (PSPC), a class of naturally occurring anthocyanins, effectively improved many features of high-fat diet (HFD)-induced NAFLD. However, whether PSPC ameliorates HFD-induced hepatocyte apoptosis has never been investigated.

**Objective:**

Here we investigated the effects of PSPC on HFD-induced hepatic apoptosis and the mechanisms underlying these effects.

**Design:**

Mice were divided into four groups: Control group, HFD group, HFD + PSPC group and PSPC group. PSPC was administered by daily oral gavage at doses of 700 mg/kg/day for 20 weeks. EX-527 (a SirT1-selective inhibitor) and *Sirt1* siRNA were used to demonstrate the Sirt1 dependence of PSPC-mediated effects on apoptotic and survival signaling pathways *in vivo* and *in vitro*.

**Results:**

Our results showed that PSPC reduced body weights, hepatic triglyceride contents, histopathological lesions and serum ALT levels in a mouse model of NAFLD induced by HFD. Furthermore, PSPC attenuated HFD-induced hepatocyte apoptosis ratio from 7.27 ± 0.92% to 1.79 ± 0.27% in mouse livers, which is insignificant compared with that of controls. Moreover, PSPC activated Sirt1 by boosting NAD^+^ level in HFD-treated mouse livers. Furthermore, PSPC promoted Sirt1-dependent suppression of P53-mediated apoptotic signaling and activation of Akt survival signaling pathway in HFD-treated mouse livers, which was confirmed by EX527 treatment. Moreover, *Sirt1* knockdown abolished these ameliorative effects of PSPC on apoptosis and P53 acetylation and protein expression in PA-treated L02 cells. Ultimately, PSPC reduced Caspase-3 activation and Bax level, and elevated the Bcl-2 level in HFD-treated mouse livers.

**Conclusion:**

PSPC protected against HFD-induced hepatic apoptosis by promoting Sirt1- dependent inhibition of p53-apoptotic pathway and facilitation of Akt survival pathway. This study indicates that PSPC is a candidate for nutritional intervention of NAFLD.

## Popular scientific summary

- Purple sweet potato color (PSPC) improved many pathological features of high-fat diet (HFD)-induced nonalcoholic fatty liver disease (NAFLD) in mice.
- This study showed that PSPC exhibited a significant ameliorative effect on HFD-induced hepatic apoptosis by restoring the NAD^+^ depletion-mediated SirT1 loss, thereby suppressing p53-apoptotic pathway and enhancing Akt survival pathway.
- Inhibition of hepatocyte apoptosis is recognized as a potential therapeutic strategy for NAFLD. Thus, PSPC is a promising candidate for nutritional intervention of NAFLD.

Nonalcoholic fatty liver disease (NAFLD), one of the most prevalent liver diseases worldwide, is closely associated with metabolic diseases, such as obesity, dyslipidemia and type 2 diabetes (T2D) (1, 2). It is well recognized that chronic intake of Western-style diet rich in fat and sugar is a major factor responsible for the development of NAFLD (1–3). However, the mechanisms contributing to the development and progression of NAFLD under high-fat (Western) diet condition have never been fully clarified.

Apoptosis, a major form of programmed cell death, is considered as a fundamental component in the pathogenesis of various liver diseases including NAFLD (4, 5). It has been established that high-fat diet (HFD) may promote the development and progression of NAFLD via inducing liver cell apoptosis (6, 7). Furthermore, recent evidences indicate that the inhibition of hepatocyte apoptosis improves NAFLD under HFD condition, which is possible to develop a potential therapeutic strategy for NAFLD (7, 8). Though liver cell apoptosis has been emerged as an important pathological mechanism of NAFLD, the mechanisms underlying HFD-induced hepatocyte apoptosis and its therapeutic potentiality remain to be investigated.

Silent mating type information regulation 2 homolog1 (SirT1), the best characterized mammalian homologs of yeast Sir2, governs a variety of physiological processes, such as aging, stress response, circadian rhythm and energy metabolism. Recently, the NAD^+^ depletion-mediated down-regulation of Sirt1 is emerging as a major contributor to the pathogenesis of various diseases including T2D, which contributes to many disorders of these diseases, such as oxidative stress, mitochondrial damage and inflammation (9, 10). It has been well demonstrated that SirT1 plays a crucial role in the suppression of cell apoptosis during multiple physiological and pathological processes, owing to its ability to deacetylate numerous substrates involving in various apoptotic and survival pathway, such as p53 and NF-κB (11, 12). Accumulating evidence indicates that SirT1 is involved in regulating hepatocyte apoptosis during NAFLD (7, 13). However, the role of SirT1 in HFD-induced hepatocyte apoptosis and the underlying mechanisms of this action need further study.

Purple sweet potato color (PSPC), a class of natural anthocyanins derived from purple sweet potato storage roots, exhibits stronger free radical scavenging activity both *in vitro* and *in vivo* (14, 15). It has been widely reported that PSPC possesses multiple physiological activities, including antioxidant, anti-inflammatory, anti-carcinogenic, anti-diabetic and hepatoprotective effects (16–20). Moreover, our previous work indicated that PSPC effectively improved many features of HFD-induced NAFLD, such as inflammation, steatosis and insulin resistance in mice (17–19). Nevertheless, whether PSPC ameliorates HFD-induced hepatocyte apoptosis has never been investigated.

It has been established that hepatocyte apoptosis contributes to the development and progression of NAFLD. SirT1 inhibits cell apoptosis under various disease conditions. Our previous work showed that PSPC effectively ameliorated hepatocyte apoptosis-mediated liver injuries in D-galactose-treated mice (21). Thus, we postulated that PSPC might improve NAFLD via ameliorating Sirt1 down-regulation-mediated hepatocyte apoptosis. This study was designed to address these issues.

## Materials and methods

### Animals and treatment

All experimental and euthanasia procedures performed in this study were approved by the Institutional Animal Care and Use Committee of Jiangsu Normal University. ICR mice (male, 8-week-old) were purchased from Hua-fu-Kang Biological Technology Co. Ltd (Beijing, China). Mice were maintained at constant temperature (23 ± 1°C) and humidity (60%), had free access to rodent food and tap water and were kept on a 12-h light/dark schedule (lights on 08:30–20:30). After acclimation for 1 week, mice were randomly divided into four groups: Control group (*n* = 8), HFD (60% of energy as fat; D12492; Research Diets, New Brunswick, NJ, USA) group (*n* = 8), HFD + PSPC group (*n* = 20) and PSPC group (*n* = 8), and received the following treatments for 20 weeks: Mice in the Control group and the PSPC group were fed a normal diet (ND, 10% of energy as fat; D12450B; Research Diets, New Brunswick, NJ, USA). Mice in the HFD group and the HFD + PSPC group were fed an HFD. PSPC was purchased from Qingdao Pengyuan Natural Pigment Research Institute (Qingdao, China). The major components of PSPC by HPLC analysis are cyanidin acyl glucosides and peonidin acyl glucosides (>90%, peonidin 3-O-(6-O-(E)-caffeoyl-2-O-β-D-glucopyranosyl-β-D-glucopyranoside) -5-O-β-D- glucoside, peonidin 3-O-(2-O-(6-O-(E)-caffeoyl-β-D-glucopyranosyl) -6-O-(E)-caffeoyl-β-D-glucopyranoside)-5-O-β-D-glucopyranoside, Peonidin3-O-(2-O-(6-O-(E)-feruloyl-β-D-glucopyranosyl)-6-O-(E)-caffeoyl-β-D-glucopyranoside)-5-O-β-D-glucopyranoside, cyanidin 3-O-(6-O-p-coumaroyl)-β-D-glucopyranoside) and the rest is other flavonoids), as described in our previous work (22).

### PSPC treatment

PSPC was dissolved in distilled water containing 0.1% Tween 80. Mice were orally gavaged with a daily 700 mg/kg/day dose of PSPC or an equal volume of distilled water containing 0.1% Tween 80. The PSPC dosage used in this study was according to our previous work (19).

### EX527 treatment

After 12 weeks of HFD treatment, 12 mice of HFD + PSPC group were randomly divided into two subgroups: HFD+PSPC group (*n* = 6) and HFD+PSPC+EX527 group (*n* = 6). Three hours before PSPC treatment, EX527 (a SirT1-selective inhibitor, SelleckBio, Houston, USA) dissolved in 99% sterile saline/1% DMSO (Sigma-Aldrich, MO, USA) was given to mice in HFD+PSPC+EX527 group by daily intraperitoneal injections (ip) at the dose of 10 mg/kg/day for 8 weeks, and the mice of HFD+PSPC group received daily ip of an equal volume of 99% sterile saline/1% dimethyl sulphoxide (DMSO).

After 20 weeks of treatment, mice were fasted overnight, anesthetized and sacrificed. The liver, epididymal fat and blood were immediately collected for experiments or stored at −80°C until analysis.

### Tissue homogenates

The preparation of liver homogenates was performed as described in our previous work (19, 23). The protein concentration was determined with a bicinchoninic acid assay kit (Pierce Biotechnology, Rockford, IL, USA) according to the manufacturer’s instructions.

### Biochemical analyses

The serum ALT activities were spectrophotometrically measured with a diagnostic kit (Jiancheng Institute of Biotechnology, Nanjing, China) following the manufacturer’s instructions.

Hepatic lipids were extracted from approximately 200 mg frozen liver samples using chloroform:methanol (2:1 v/v) solution, as described by Folch and Lees (24) and resuspended in PBS containing 5% Triton X-100 (Amresco, Solon, OH, USA). The serum sample and hepatic lipid extraction solution were used to determine TG levels using the corresponding LabAssay kit (Wako Chemicals, Richmond, VA, USA) according to the manufacturer’s instructions.

### Liver slice collection and histopathological analysis

Liver slice collection and hematoxylin-eosin staining were performed according to the protocols described in our previous work (19, 23). The liver sections stained with HE (Sigma-Aldrich, St. Louis, MO, USA) were examined using a Zeiss Axioskop 40 microscope (Carl Zeiss, Göttingen, Germany).

### Terminal deoxyribonucleotidyl transferase-mediated dUTP-digoxigenin nick-end labeling assay

Terminal deoxyribonucleotidyl transferase-mediated dUTP-digoxigenin nick-end labeling (TUNEL) staining was performed to assess apoptosis with an In Situ Cell Death Detection Kit, Flourescein (Roche, Indianapolis, IN, USA) according to the instructions of the manufacturer. A double-staining technique was used; that is, TUNEL staining was used for the apoptotic hepatocyte nuclei, and ProLong^®^ Gold containing 4, 6-diamidino-2-phenylindole (Invitrogen, Carlsbad, CA, USA) staining was used for all hepatocytes. Stained specimens were captured using a Zeiss Axioskop 40 microscope, and images were taken with a CCD camera (CoolSNAP Color; Photometrics, Roper Scientific). Apoptosis was quantified by determining the percentages of TUNEL-positive cells in 10 random microscopic fields at 200× magnification per specimen.

### NAD^+^ assay

NAD^+^ levels were measured using EnzyChromTM NAD+/NADH Assay kit (BioAssay Systems, Hayward, CA, USA) following the manufacturer’s instructions. The NAD levels were expressed as pmol/mg liver.

### SirT1 activity determination

SirT1 activity was measured using a SirT1 Fluorometric Drug Discovery Kit (ENZO Life Sciences International, Inc. PA, USA) according to the manufacturer’s instructions. This assay uses a peptide containing human p53 amino acids 379–382 (Arg-His-Lys-Lys(Ac)) as a substrate. SirT1 activity is proportional to the amount of Lys-382 deacetylation. Fluorescence was assessed in a Molecular Devices M2 plate reader (Molecular Devices Corporation, Menlo Park, CA, USA) with an excitation wavelength of 360 nm and an emission wavelength of 460 nm. Changes in SirT1 activity in livers were calculated against the mean value of SirT1 activity in control liver and expressed as percent of control.

### Immunofluorescence staining

The preparation of frozen sections and immunofluorescence staining was performed as described previously (19, 23). The liver sections were incubated with the primary antibody (rabbit anti-4-HNE antibody, 1:100, Alpha Diagnostics, San Antonio, TX, USA) overnight at 4°C. After a washing with phosphate-buffered saline, the liver sections were incubated with Texas Red-conjugated anti-rabbit IgG (1:200, Vector Laboratories, Inc., Burlingame, CA, USA).

### ROS assay

Reactive oxygen species (ROS) assay was performed based on the oxidation of 2’, 7’-dichlorodihydrofluorescein diacetate (H2-DCF-DA) to 2’, 7’-dichlorofluorescein (DCF) as previously described [19,23]. The conversion of H2-DCF-DA to the fluorescent product DCF was measured with a Molecular Devices M2 plate reader (Molecular Devices Corporation) (excitation at 484 nm and emission at 530 nm). ROS formation was quantified from a DCF standard curve. Data are calculated as pmol DCF formed/min/mg protein.

### GSH assay

The levels of Glutathione (GSH) in the hepatic supernatants were determined according to the protocols of a commercially available GSH assay kit (Cayman Chemical, Ann Arbor, MI, USA). After reaction with 5,5-dithiobes-(2-ni-trobenzoic acid) (DTNB), the GSH contents were measured by a spectrophotometer (Shimadzu UV-2501PC) at 405 nm. The GSH contents were calculated as the contents (μmol GSH) per mg protein.

### Cell culture and treatments

The human normal liver cell line L02 cells were obtained from Shanghai Cell Bank of the Chinese Academy of Sciences (Shanghai, China). L02 cells were cultured in Dulbecco's modified eagle medium (DMEM; Gibco, Carlsbad, CA, USA) cell culture medium supplemented with 10% heat-inactivated fetal bovine serum (FBS; Gibco, Carlsbad, CA, USA), 100 units/mL penicillin and 100 μg/mL streptomycin. The cells were incubated in a humidified cell incubator at 37°C, 5% CO_2_.

*Sirt1* siRNA (sc-40986) and control siRNA (sc-37007) were obtained from Santa Cruz Biotechnology (Dallas, TX, USA). The L02 cells were seeded in 6-well plates for 24 h and then transfected with *Sirt1* siRNA or control siRNA using Lipofectamine 3000 reagent (Invitrogen Inc., CA, USA) according to the manufacturer's instructions. Forty-eight hours after transfection, L02 cells were treated with PSPC (50 μg/mL, according to our previous study (25) for 4 h, and then treated with palmitic acid (PA) or bovine serum albumin (BSA) for 48 h.

PA powder (Sigma-Aldrich, St. Louis, MO, USA) was dissolved in 0.1 M NaOH at 70°C by water bath, then added to a 10% solution of fatty acid free BSA and filtered on a 0.22-μm filter, yielded a 10 mM stock solution. The PA stock solution was added to L02 cells at a final concentration of 500 μM.

### Caspase-3 activity assay

Hepatic caspase-3 activity was determined with a caspase-3 cellular activity assay kit (Calbiochem, San Diego, CA, USA) following the manufacturer’s instructions. Caspase-3 activity was quantified by measuring the colorimetric release of chromophore ρnitroanilide (ρNA) that is cleaved from the substrate acetyl-Asp-Glu-Val-Asp p-nitroanilide (Ac-DEVD-pNA). The ρNA level was determined at 405 nm using a Molecular Devices M2 plate reader. Caspase-3 activity is calculated as pmol/min/mg protein.

### Western blot analysis

The western blot analyses were performed as described in our previous work (19, 23). The primary antibodies were as follows: rabbit anti-SirT1 and rabbit anti-AC-P53 (Lys373, Lys382) antibodies (Millipore, Billerica, MA, USA); rabbit anti-P-Akt (Ser473), rabbit anti-Akt, rabbit anti-P-GSK-3β (Ser9), rabbit anti-GSK-3β, rabbit anti-cleaved-caspase-3, mouse anti-P53 and rabbit anti-β-Actin antibodies (Cell Signaling Technology, Beverly, MA, USA); mouse anti-Bax and mouse anti-Bcl-2 antibodies (BD Biosciences, San Diego, CA, USA) and rabbit anti-P21 antibody (Abcam, Cambridge, UK). After washing, proteins were detected using horseradish peroxidase (HRP)-conjugated anti-rabbit and HRP-conjugated anti-mouse secondary antibodies (Cell Signaling Technology, Beverly, MA, USA). The optical density (OD) values of the immunoblot bands were quantified with Scion Image analysis software (Scion Corp., Frederick, MD, USA). The OD values were normalized using appropriate internal controls (optical density detected protein/optical density internal control).

### Statistical analysis

All statistical analysis was performed using SPSS version 11.5. All the data were analyzed with a one-way ANOVA followed by Tukey’s Honestly Significant Difference (HSD) post hoc test (more than two groups) and Student’s *t*-test (two groups). Data were expressed as means ± standard deviation (SD). Statistical significance was set at *P* < 0.05.

## Results

### PSPC attenuates hepatocyte apoptosis in HFD-induced NAFLD mouse model

**Fig. 1:**
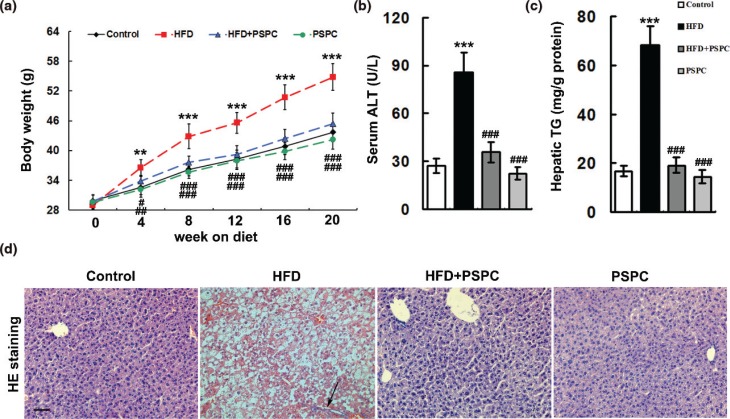
Effects of PSPC on the pathological features of NAFLD in HFD-treated mice. (a) Total body weight in all treated groups (*n* = 8). (b) Serum ALT activities in all treated groups (*n* = 5). (c) Hepatic TG levels in all treated groups (*n* = 5). (d) H&E staining of liver sections (*n* = 5). The arrow indicates congregated leucocytes and migratory leucocytes. Magnification 200x. All of the values are expressed as the mean ± SD. **P < 0.01, ***P < 0.001 versus the control group; #*P* < 0.05, ##*P* < 0.01, ###*P* < 0.001 versus the HFD group.

After 20 weeks of feeding, the body weights of HFD-fed mice were significantly higher (54.8 ± 2.66 g) than those of ND-fed mice (43.7 ± 1.9 g) (Fig. 1a). The notably increased levels of serum alanine aminotransferase (ALT, 85.99 ± 14.75 U/L) and hepatic triglycerides (TG, 68.4 ± 7.47 mg/g protein) were found in HFD-fed mice compared to those of controls (ALT: 27.22 ± 4.53 U/L; TG: 16.7 ± 2.25 mg/g protein) (Fig. 1b and c). Hematoxylin and eosin (H&E) staining showed that HFD caused the hepatocyte hypertrophy and vacuolization and inflammatory cell infiltration in mouse livers, further confirming the occurrence of NAFLD (Fig. 1d). Interestingly, PSPC strikingly reversed these pathological liver injuries and NAFLD-related parameters in HFD-treated mice (body weights: 45.4 ± 2.13 g; ALT: 35.79 ± 6.55 U/L; TG: 19.17 ± 3.11 mg/g protein) (Fig. 1). No marked differences in body weights, hepatic fat accumulation and liver injuries were observed among the HFD+PSPC, PSPC and the vehicle control groups.

**Fig. 2:**
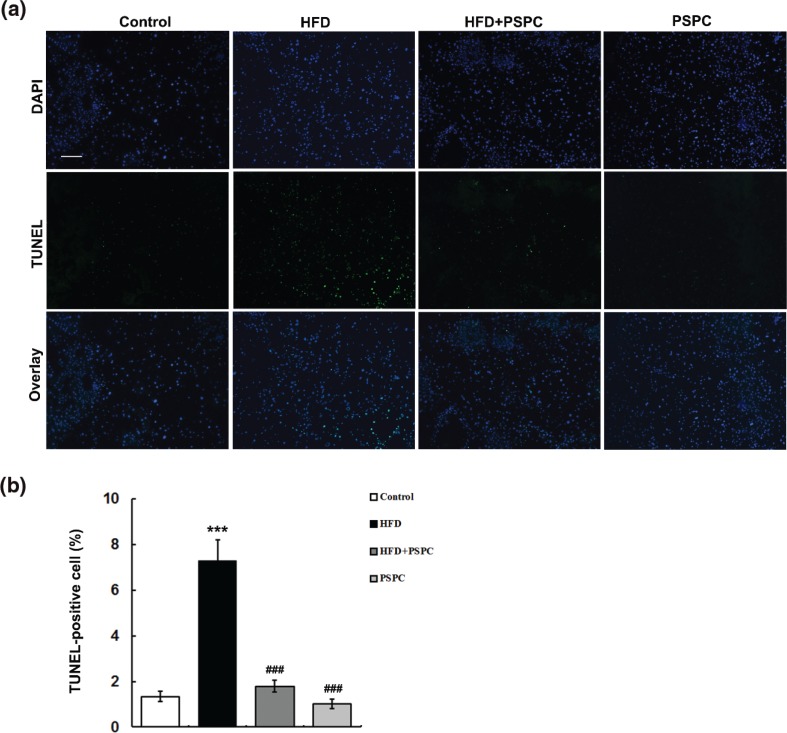
Effects of PSPC on hepatocyte apoptosis in HFD-treated mice (*n* = 5). (a) Representative micrographs of hepatic TUNEL staining from mice in different treatment groups. Magnification, 100x. (b) Quantitative results of hepatic TUNEL staining. All of the values are expressed as mean ± SD. ***P < 0.001 versus the control group; ###*P* < 0.001 versus HFD group.

The results of TUNEL assay showed that HFD significantly elevated the percentages of hepatocytes undergoing apoptosis in the mouse livers (7.27 ± 0.92%) compared to those of controls (1.36 ± 0.23%) (Fig. 2). Interestingly, PSPC markedly decreased the hepatocyte apoptosis ratio to 1.79 ± 0.27% in HFD-treated mice (Fig. 2). There was no evident difference in hepatocyte apoptosis among the HFD+PSPC, PSPC and the control groups. These results indicated that PSPC protected against hepatocyte apoptosis in HFD-induced NAFLD mouse model.

### PSPC promotes Sirt1 activation in HFD-treated mouse livers

**Fig. 3:**
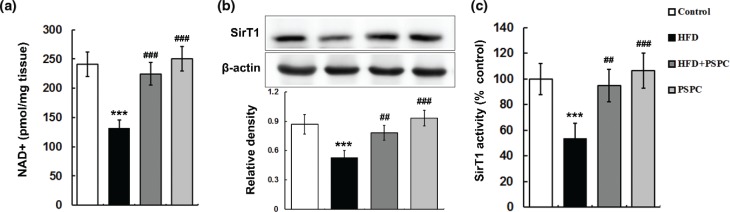
Effects of PSPC on Sirt1 activation in HFD-treated mouse livers (*n* = 5). (a) NAD^+^ levels in mouse livers. (b) Immunoblotting and densitometry of SirT1 in mouse livers. (c) SirT1 activities in mouse livers. All of the values are expressed as the mean ± SD. ***P < 0.001 versus the control group; ##*P* < 0.01, ###*P* < 0.001 versus the HFD group.

HFD feeding caused an obvious diminution of NAD^+^ levels in mouse livers (131.29 ± 14 pmol/mg tissue) compared to those of controls (241.38 ± 20.98 pmol/mg tissue) (Fig. 3a). Interestingly, PSPC effectively renewed the NAD^+^ level (224.49 ± 19.41 pmol/mg tissue) in HFD-fed mouse livers (Fig. 3a). A remarkable reduction in protein expression of SirT1 as well as its activity (53.4 ± 11.84%) was observed in HFD-fed mouse livers compared to those of controls (Fig. 3b and c). Interestingly, PSPC dramatically restored SirT1 protein expression and activity (94.6 ± 12.7%) in the livers of HFD-fed mice (Fig. 3b and c). There were no significant differences in NAD^+^ and SirT1 levels among the HFD + PSPC, PSPC and control groups.

**Fig. 4:**
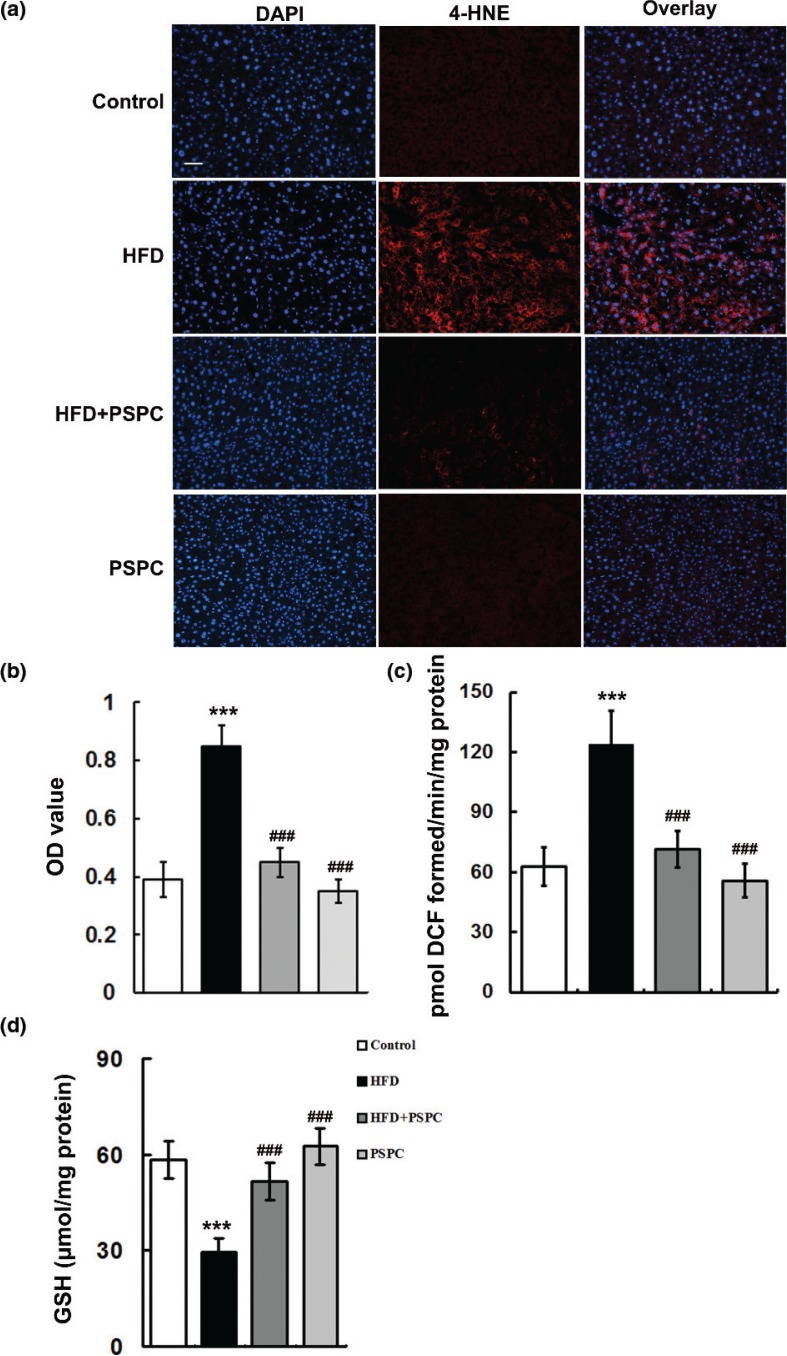
Effects of PSPC on oxidative stress in HFD-treated mouse livers (*n* = 5). (a) 4-HNE immunofluorescence staining. Magnification 200x. (b) 4-HNE fluorescence intensity was measured as the mean OD value. (c) ROS productions in mouse livers. (d) GSH levels in mouse livers. All of the values are expressed as the mean ± SD. ***P < 0.001 versus the control group; ###*P* < 0.001 versus the HFD group.

It is well established that chronic oxidative stress diminishes Sirt1 levels in various tissues. Thus, we determined the levels of oxidative stress markers including 4-hydroxynonenal (4-HNE, a marker of lipid peroxidation) and ROS in mouse livers. Our results showed there was a remarkable oxidative stress characterized by significantly increased levels of 4-HNE (0.85 ± 0.07) and ROS (123.62 ± 17.38 pmol DCF formed/min/mg protein), notably diminished GSH level (29.28 ± 4.41 μmol/mg protein) in HFD-treated mouse livers compared to those of controls (4-HNE: 0.39 ± 0.06; ROS: 62.81 ± 9.51 pmol DCF formed/min/mg protein; GSH: 58.42 ± 5.87 μmol/mg protein) (Fig. 4). Interestingly, PSPC obviously reduced the levels of 4-HNE (0.46 ± 0.05) and ROS (71.35 ± 7.12 pmol DCF formed/min/mg protein), while restored GSH content (51.78 ± 5.94 μmol/mg protein) in the livers of HFD-treated mice (Fig. 4). No remarkable differences were found in hepatic redox status among the HFD+PSPC, PSPC and the vehicle control group. These results suggested that PSPC might strikingly promote SirT1 activation by inhibiting oxidative stress in the HFD-treated mouse livers.

### PSPC suppresses P53-mediated apoptosis signaling in HFD-treated mouse livers

**Fig. 5:**
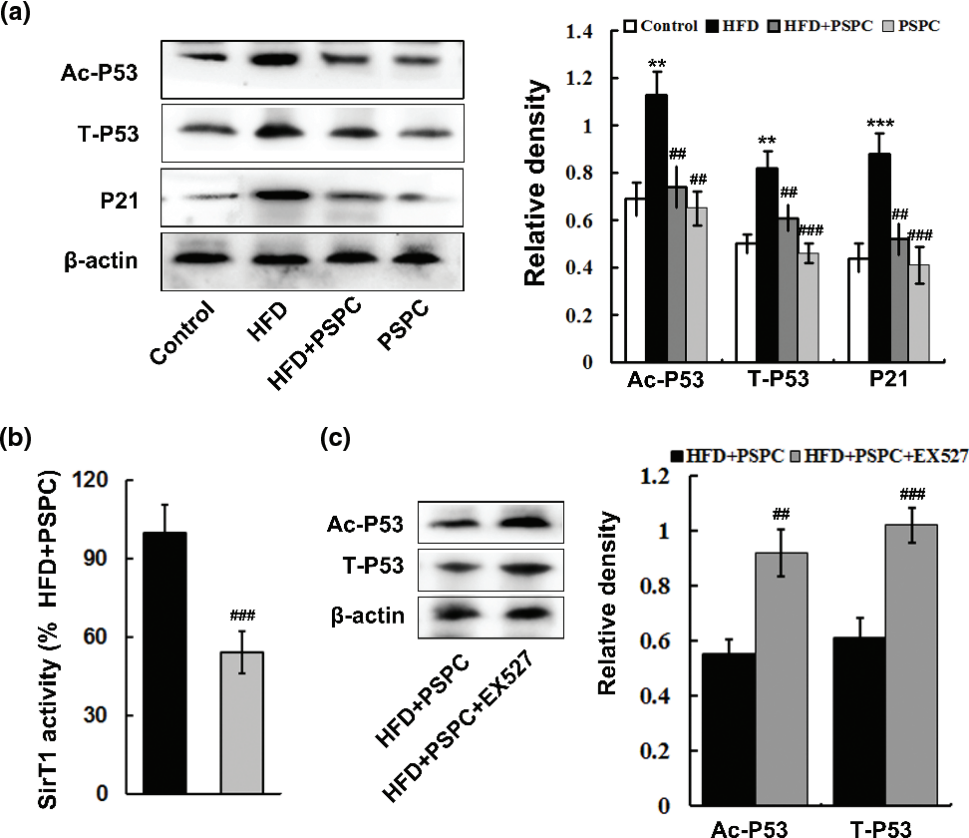
Effects of PSPC on P53-mediated apoptosis signaling in HFD-treated mouse livers. (a) Immunoblotting and densitometry of AC-P53, T-P53 and P21 in mouse livers (*n* = 3). (b) SirT1 activities in mouse livers (*n* = 5). (c) Immunoblotting and densitometry of AC-P53 and T-P53 in mouse livers (*n* = 3). All of the values are expressed as the mean ± SD. **P < 0.01, ***P < 0.001 versus the control group; ##*P* < 0.01, ###*P* < 0.001 versus the HFD group.

There were markedly elevated protein expressions of AC-P53 (Lys373, Lys382) and total P53, which resulted in a consequent increase of P21 protein expression in HFD-fed mouse livers (Fig. 5a). Interestingly, PSPC dramatically decreased the protein levels of AC-P53, T-P53 and P21 in HFD-fed mouse livers (Fig. 5a). No obvious differences in those protein expressions were found among the HFD + PSPC, PSPC and control groups.

To investigate whether PSPC inhibited P53-mediated apoptosis signaling by restoring SirT1 level, we blocked SirT1 activity by EX527 (a selective inhibitor of SirT1) in mouse livers. EX527 markedly abated SirT1 activities (54.2 ± 8.04%) of mouse livers in HFD+PSPC group (Fig. 5b). Moreover, PSPC-mediated inhibition of P53 acetylation and protein expression was markedly blunted by EX527 in mouse livers (Fig. 5c).

**Fig. 6:**
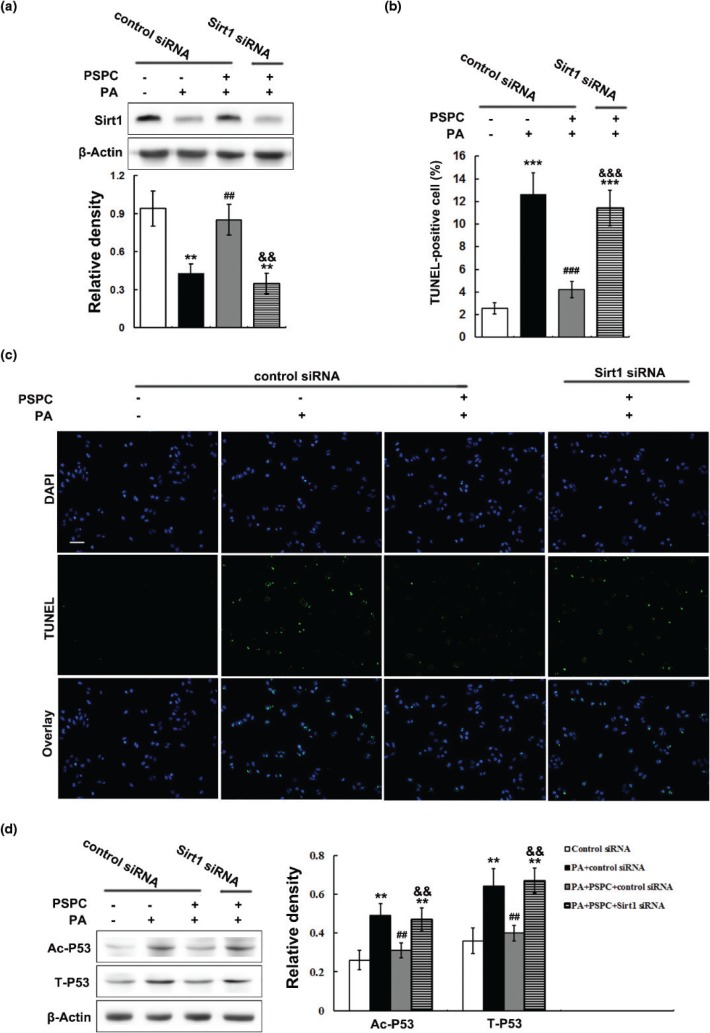
Effects of *Sirt1* knockdown on PSPC-mediated suppressions of apoptosis and P53 acetylation and protein expression in L02 cells. (a) Immunoblotting and densitometry of SirT1 in L02 cells. (b) Quantitative results of TUNEL staining in L02 cells. (c) Representative micrographs of TUNEL staining of L02 cells in different treatment groups. Magnification, 200x. All of the values are expressed as mean ± SD. **P < 0.01, ***P < 0.001 versus the control siRNA group; ##*P* < 0.01, ###*P* < 0.001 versus PA+control siRNA group; &&*P* < 0.01, &&&*P* < 0.001 versus PA+PSPC+control siRNA group.

*In vitro* experiments using normal human hepatic cell line L02 were conducted to further confirm that Sirt1 is responsible for PSPC-mediated suppression of apoptosis via decreasing P53 acetylation and protein expression. The cells of PA+PSPC group were treated with *Sirt1* siRNA to knockdown *Sirt1*. PA treatment notably reduced Sirt1 protein expression, which was effectively restored by PSPC treatment in L02 cells (Fig. 6a). Sirt1 level was decreased by 59.31% in the cells of PA+PSPC group treated with *Sirt1* siRNA as compared with that of control siRNA treatment (Fig. 6a), which conformed the efficiency of siRNA-mediated *Sirt1* knockdown. PA markedly elevated the percentages of apoptotic L02 cells (12.62 ± 1.89%) compared to that of control cells (2.57 ± 0.52%), which was largely inhibited by PSPC treatment (4.22 ± 0.7%) (Fig. 6b and c). Moreover, PA notably increased P53 acetylation and protein expression in L02 cells (Fig. 6d). Interestingly, PSPC treatment significantly diminished P53 acetylation and protein expression in PA-treated L02 cells (Fig. 6d). However, *Sirt1* knockdown dramatically abolished these ameliorative effects of PSPC on cell apoptosis (11.7 ± 1.57%) and P53 acetylation and protein expression in PA-treated L02 cells (Fig. 6b–d).

Taken together, these results indicated that PSPC inhibited P53-mediated apoptotic signaling through up-regulating SirT1 level in HFD-fed mouse livers.

### PSPC renews Akt survival signaling pathway in HFD-treated mouse livers

**Fig. 7:**
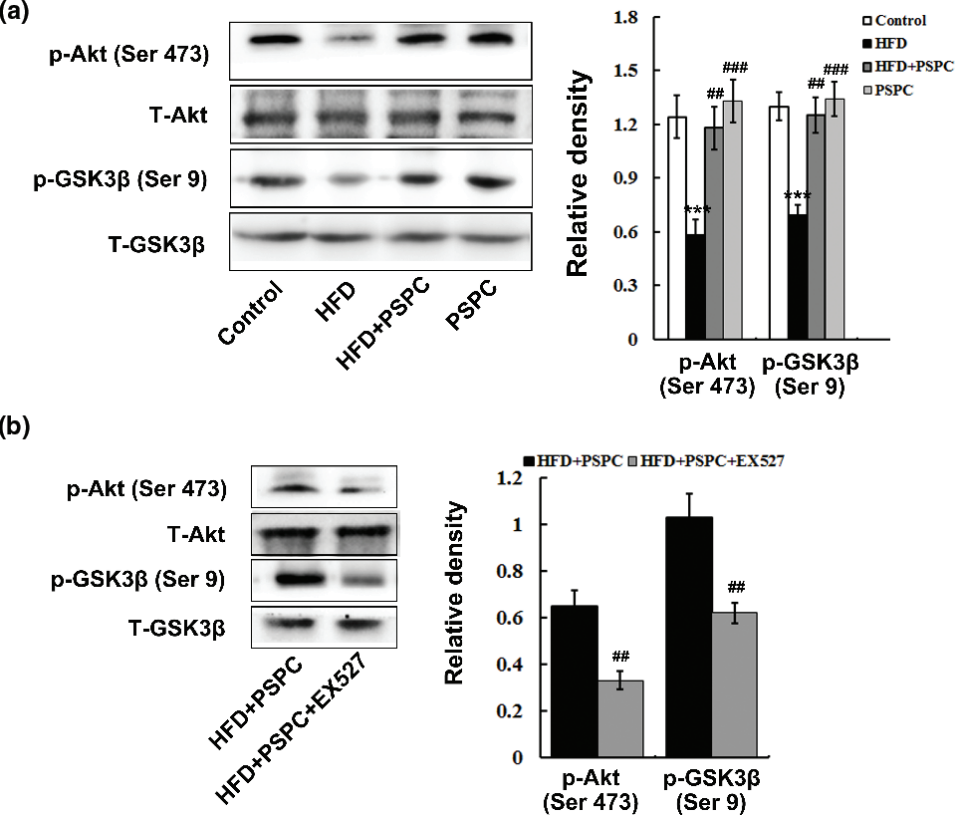
Effects of PSPC on Akt survival pathway in HFD-treated mouse livers (*n* = 3). (a and b) Immunoblotting and densitometry analysis of p-Akt and p-GSK-3β in mouse livers. All of the values are expressed as the mean ± SD. **P < 0.01, ***P < 0.001 versus the control group; ##*P* < 0.01, ###*P* < 0.001 versus the HFD group.

HFD feeding significantly abated the Akt (ser-473) phosphorylation levels in mouse livers (Fig. 7a), suggesting a notable decrease in Akt activation. HFD consequently enhanced GSK-3β signaling as evidenced by reducing GSK-3β (ser-9) phosphorylation levels in mouse livers (Fig. 7a). Interestingly, PSPC markedly increased the levels of Akt (ser-473) phosphorylation, resulting in the elevation of GSK-3β (ser-9) phosphorylation in the HFD-fed mouse livers (Fig. 7a). There were no visible differences among the HFD+PSPC, PSPC and the control groups. However, EX527 dramatically restrained the PSPC-mediated Akt activation and GSK-3β inhibition in HFD-fed mouse livers (Fig. 7b). These results showed that PSPC renewed Akt survival signaling pathway by elevating SirT1 level in HFD-fed mouse livers.

### PSPC ameliorates apoptosis-related protein levels in HFD-treated mouse livers

**Fig. 8:**
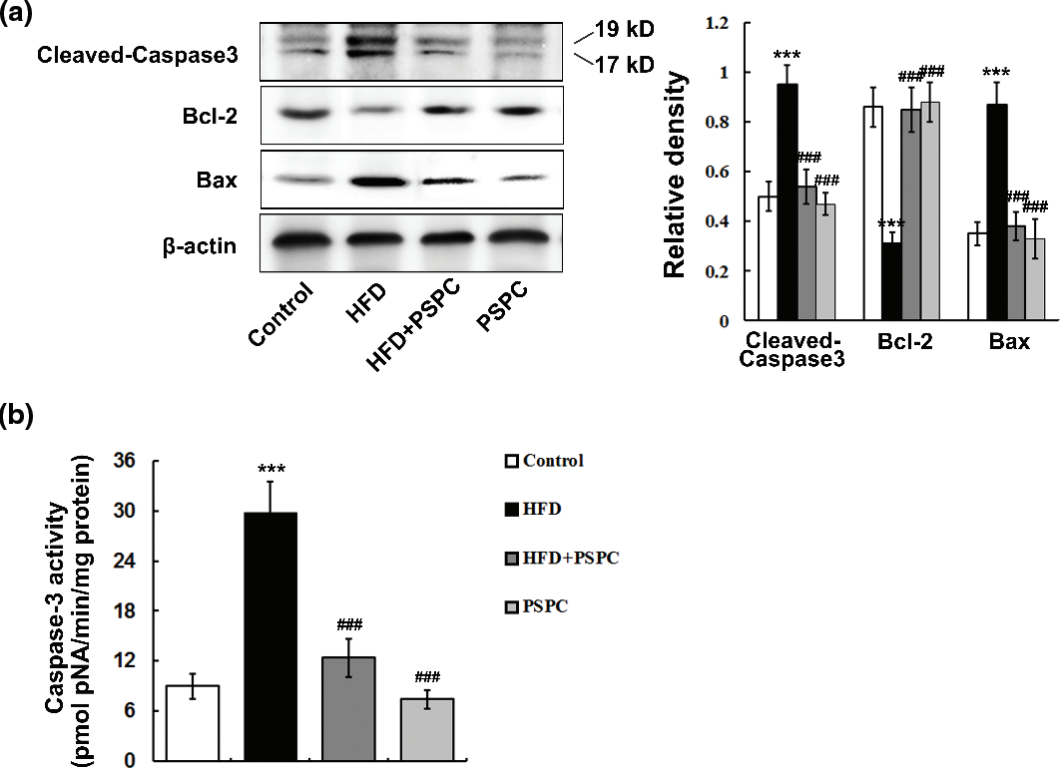
Effects of PSPC on apoptosis-related protein levels in HFD-treated mouse livers. (a) Immunoblotting and densitometry analysis of apoptosis-related protein in HFD-treated mouse livers (*n* = 3). (b) Caspase-3 activities in mouse livers (*n* = 5). All of the values are expressed as the mean ± SD. **P < 0.01, ***P < 0.001 versus the control group; ##*P* < 0.01, ###*P* < 0.001 versus the HFD group.

A strikingly elevated protein expressions of cleaved-Caspase-3 and Bax, as well as an obviously decreased Bcl-2 levels, were observed in HFD-treated mouse livers (Fig. 8a). Moreover, the activities of Caspase-3 were markedly increased in the livers of HFD-fed mice (29.8 ± 3.7 pmol/min/mg protein) compared to those of control mice (9 ± 1.58 pmol/min/mg protein) (Fig. 8b). Interestingly, PSPC notably reduced the protein expressions of cleaved-Caspase-3 and Bax and Caspase-3 activities (12.4 ± 2.3 pmol/min/mg protein), and elevated the Bcl-2 level in HFD-treated mouse livers (Fig. 8). No evident differences in those apoptosis-related protein levels were found among the HFD + PSPC, PSPC and control groups. These results indicated that PSPC ameliorated apoptosis-related protein levels in HFD-treated mouse livers.

## Discussion

NAFLD, the hepatic manifestation of metabolic syndrome, currently becomes a worldwide health concern because its incidence is rising at an alarming rate. However, the contributing mechanism in the development of NAFLD has never been fully clarified, and there are still no confirmed clinical treatment strategies for this disease. Substantial evidences have indicated that natural plant products ameliorate various symptoms of NAFLD, including inflammation and the disorders of glucose and lipid metabolism (26–28). In the present study, our results revealed that PSPC effectively attenuated hepatocyte apoptosis by restoring the NAD^+^ depletion-mediated SirT1 loss, thereby suppressing p53-apoptotic pathway and enhancing Akt survival pathway, ultimately improving NAFLD. Moreover, in this study, PSPC exhibited a slight (insignificant) inhibitory effect on apoptotic pathways and a slight (insignificant) facilitative effect on Sirt1 activation and cell survival pathways in the livers of ND-treated mice. Our results suggested that PSPC didn’t lead to adverse effects on normal mice at present dosage. Thus, this study provides novel mechanistic insights into the pathogenesis of NAFLD and potential therapeutic targets of PSPC for NAFLD and metabolic syndrome.

Hepatocyte apoptosis is a prominent pathologic feature of NAFLD and plays an important role in the progression from simply steatosis to non-alcoholic steatohepatitis (NASH) (4–7). It has been well established that elevated circulating free fatty acids (FFAs) promote hepatic lipid accumulation under the HFD and obesity conditions, leading to lipotoxic liver injury, which activates endoplasmic reticulum (ER) stress and mitochondrial dysfunction-dependent apoptotic signaling, resulting in hepatic apoptosis (29, 30). In the present study, our data showed that HFD induced marked hepatic fat accumulation, augmented apoptosis-related protein levels and the percentages of hepatocytes undergoing apoptosis, indicating the occurrence of hepatic lipoapoptosis. Accumulating evidence suggests that in contrast to their apoptotic effects on cancer cells, naturally occurring polyphenols protect normal tissue cells from apoptosis under a variety of pathological conditions (31–33). In this study, PSPC notably attenuated hepatic fat accumulation and hepatocyte apoptosis. Our findings indicated that PSPC might suppress hepatic apoptosis via its inhibitory effects on hepatic fat accumulation.

It has been well established that Sirt1 plays a critical role in apoptosis inhibition under various pathological conditions (11, 12, 34). A growing body of evidence suggests that Sirt1 is down-regulated in diverse tissue injuries and diseases, which promotes apoptosis to cause tissue damage (11, 12, 34). In this study, HFD remarkably reduced the protein expression of SirT1 as well as its activity in mouse livers, indicating that HFD-induced hepatic apoptosis may be associated with the down-regulation of Sirt1. It has been widely reported that some naturally occurring polyphenols, such as resveratrol, are Sirt1 activators, which exhibit beneficial effects on a variety of diseases including NAFLD (12, 35). Our previous study also demonstrated that troxerutin, a flavonoid, markedly renewed Sirt1 levels to protect mouse liver from HFD-induced steatosis (23). In the present study, our results showed that PSPC significantly elevated the SirT1 level in the livers of HFD-fed mice. Moreover, *Sirt1* knockdown by siRNAs markedly abrogated the inhibitory effect of PSPC on PA-induced apoptosis in L02 cells. Consistent with these studies (12, 23, 35), our results revealed that PSPC might exhibit inhibitory effects on HFD-induced hepatic apoptosis via restoring Sirt1 level.

P53, a critical tumour suppressor, is activated by many stress stimuli, such as DNA damage and oxidative stress, thereby promoting the transcription of pro-apoptotic proteins to induce apoptosis (36, 37). It is reported that up-regulated P53 facilitates steatosis and subsequent lipotoxic liver injury including apoptosis under HFD condition (36, 37). In this study, HFD markedly elevated the expression of P53 and its acetylation at Lys379 in the mouse livers. P53 is identified as a key downstream target of Sirt1, and its transcriptional activity was restrained by SirT1-dependent deacetylation (12, 38). Therefore, our findings indicated that HFD might trigger p53-dependent hepatic apoptosis by down-regulating Sirt1. Akt is a pivotal survival kinase that mediates a variety of cell survival signaling pathways, such as inhibition of BCL-2 family pro-apoptotic member, to oppose apoptosis (39, 40). GSK-3β is a well-identified inhibitory target of Akt which plays a key role in the promotion of apoptosis (41, 42). In the present study, our results showed that a notable decrease in Akt activation and consequently enhanced GSK-3β signaling were found in HFD-treated mouse livers. Substantial evidences indicate that Sirt1 can deacetylate and activate Akt/ GSK-3β pathway to regulate various physiological and pathological processes including apoptosis (43). Thus, our data suggested that HFD might induce hepatic apoptosis via suppressing SirT1-mediated activation of Akt survival signaling. It has been well established that naturally occurring polyphenols suppress P53 activation and enhance Akt survival signaling to protect normal tissue cells against apoptosis (44, 45). In this study, PSPC remarkably decreased P53 activation and increased Akt survival signaling in HFD-fed mouse livers, which was dramatically abolished by Sirt1 inhibitor EX527. Furthermore, *Sirt1* knockdown significantly restrained the PSPC-mediated P53 inhibition and Akt activation in PA-treated L02 cells. Collectively, our findings revealed that PSPC might protect mouse liver from HFD-induced apoptosis through promoting Sirt1-mediated P53 inhibition and Akt activation.

Increasing evidences have highlighted the beneficial effects of NAD^+^ on diet-induced disorders of glucose and lipid metabolism (46, 47). NAD^+^ depletion plays an important role in the development and progression of many diseases (48, 49). Moreover, recent evidences suggest that naturally occurring polyphenols exhibit their ameliorative effects on metabolic syndrome including NAFLD by boosting NAD^+^ level (23, 50). In the present study, PSPC significantly restored the NAD^+^ level in the livers of HFD-treated mouse. Oxidative stress is widely reported to be responsible for NAD^+^ depletion by impairing NAD^+^ biosynthesis and elevating NAD^+^ breakdown under various pathological conditions including metabolic syndrome (51, 52). Consistent with our previous works (17–19), our present results showed that PSPC markedly abated HFD-induced oxidative stress in mouse livers. Therefore, our findings indicated that PSPC might increase NAD^+^ level to enhance Sirt1 activation via its antioxidant activity in the HFD-treated mouse livers.

## Conclusions

In summary, our findings highlighted the significant beneficial effects of PSPC on hepatic apoptosis in HFD-induced NAFLD. Our results showed that PSPC abated oxidative stress to restore NAD^+^ level, consequently promoting Sirt1 activation, thereby inhibiting p53-apoptotic pathway and enhancing Akt survival pathway, ultimately attenuating apoptosis in the HFD-treated mouse livers. This study provides novel mechanistic insights into NAFLD pathogenesis and indicates that PSPC is a candidate for nutritional intervention of NAFLD.

## Conflict of interest and funding

The authors declare no potential conflicts of interest. This work is supported by the National Natural Science Foundation of China (81570531, 81571055, 81672731), the Priority Academic Program Development of Jiangsu Higher Education Institutions (PAPD), the Scientific Research Support Project for Teachers with Doctor’s Degrees (15XLR005), Postgraduate Research & Practice Innovation Program of Jiangsu Province (KYCX18_2133, KYCX17_1623) and Undergraduate Student Innovation Program ofJiangsu Province (201810320086Z, 201610320054Z).

## Disclaimers

The views expressed in this article are the authors’ own and not an official position of the institution or funder.
